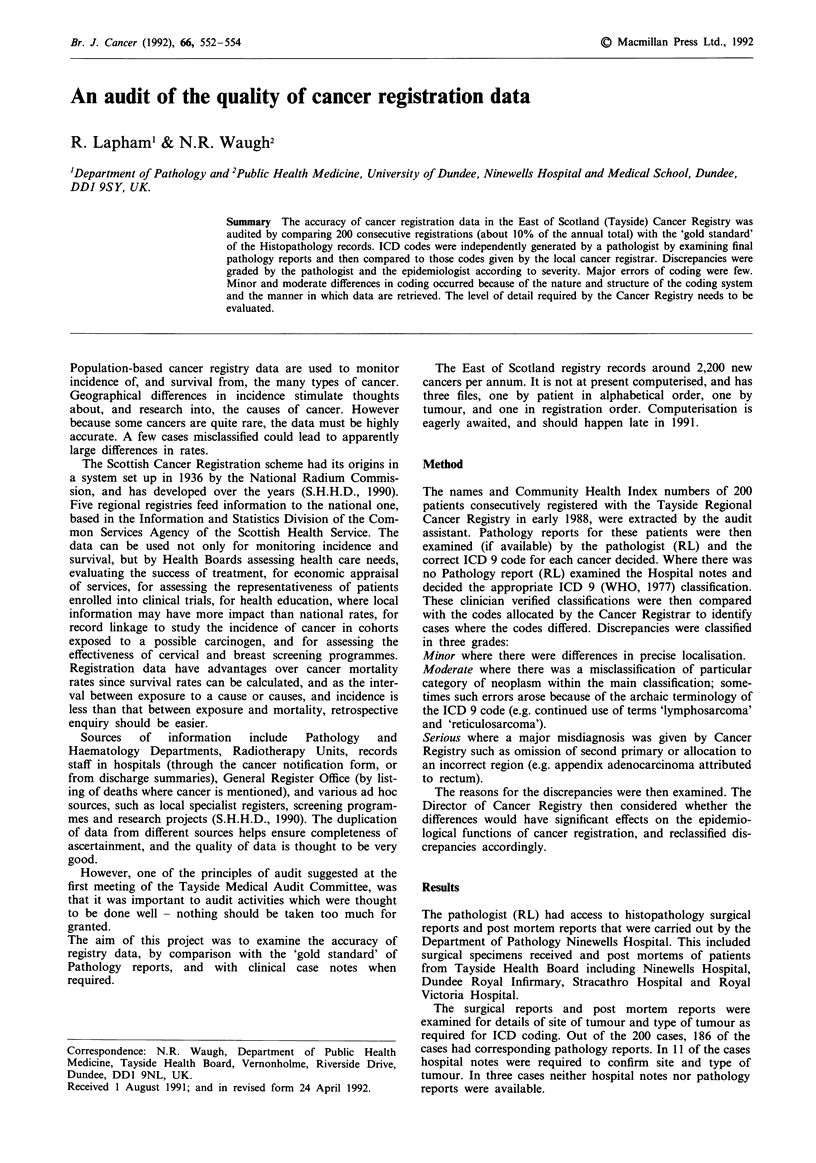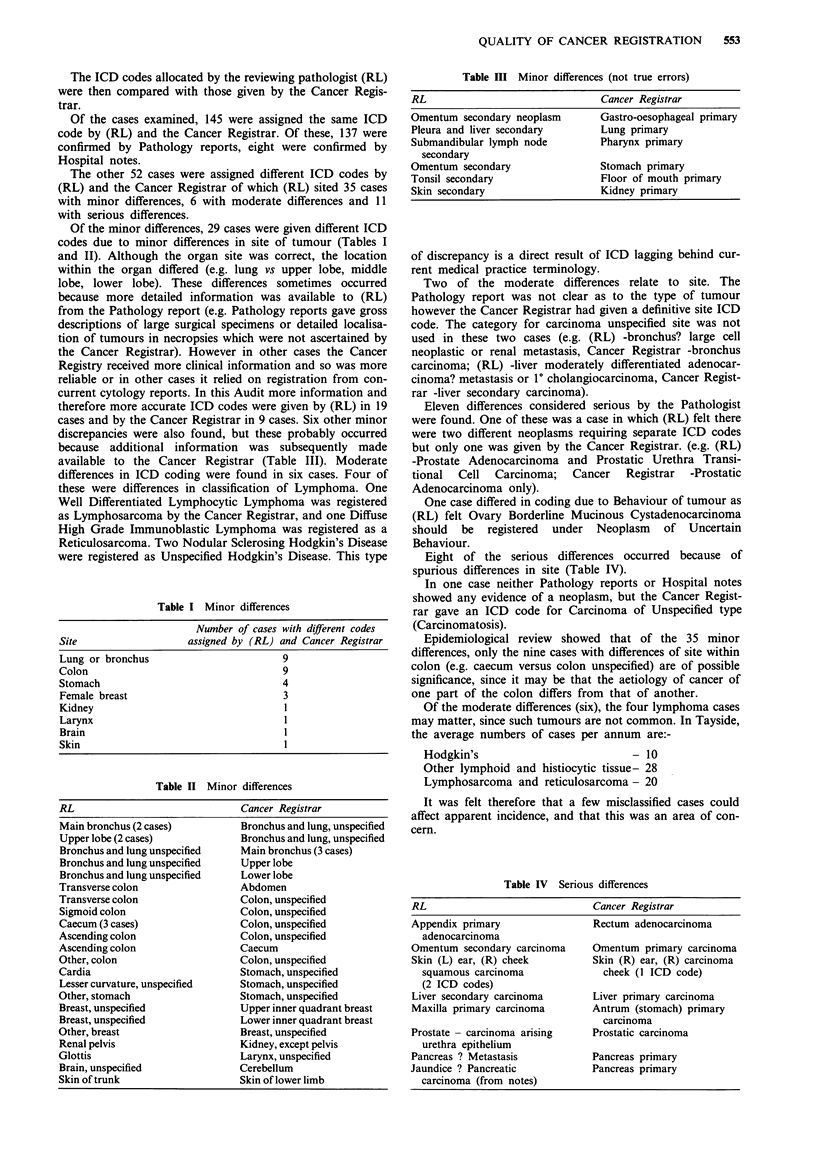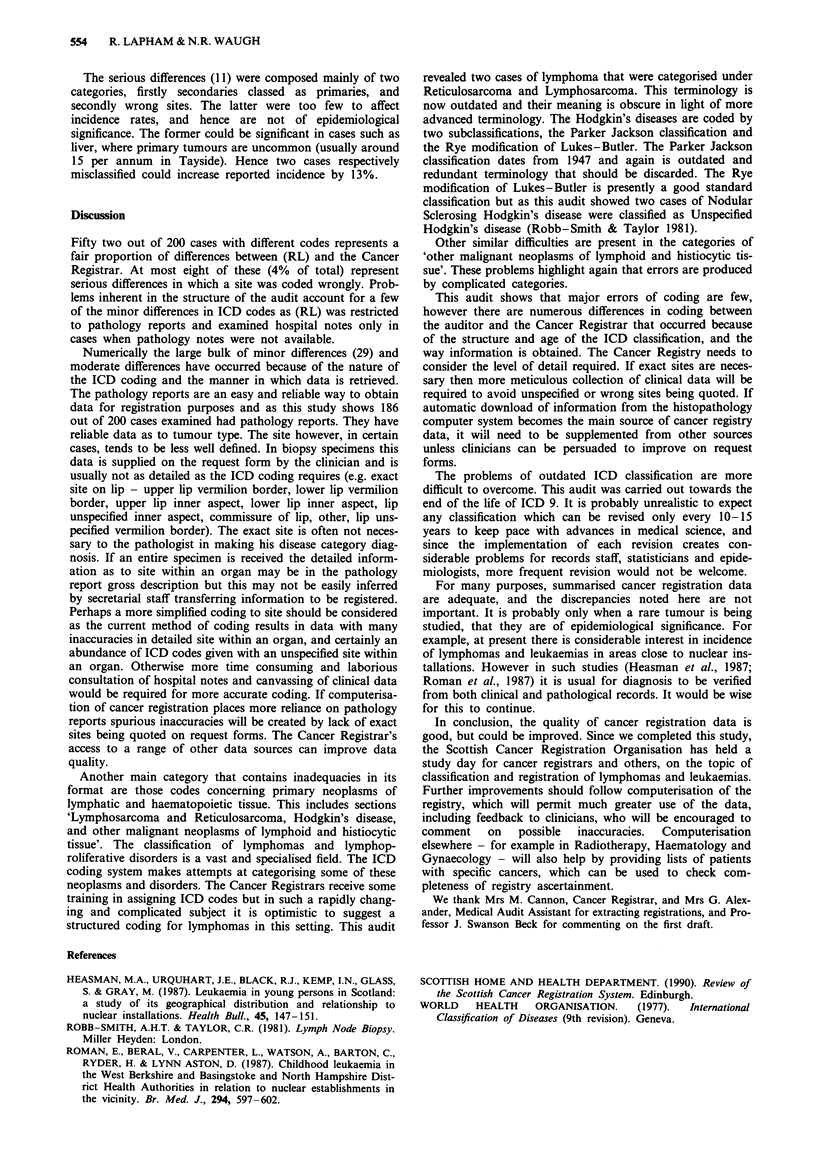# An audit of the quality of cancer registration data.

**DOI:** 10.1038/bjc.1992.312

**Published:** 1992-09

**Authors:** R. Lapham, N. R. Waugh

**Affiliations:** Department of Pathology, University of Dundee, Ninewells Hospital and Medical School, UK.

## Abstract

The accuracy of cancer registration data in the East of Scotland (Tayside) Cancer Registry was audited by comparing 200 consecutive registrations (about 10% of the annual total) with the 'gold standard' of the Histopathology records. ICD codes were independently generated by a pathologist by examining final pathology reports and then compared to those codes given by the local cancer registrar. Discrepancies were graded by the pathologist and the epidemiologist according to severity. Major errors of coding were few. Minor and moderate differences in coding occurred because of the nature and structure of the coding system and the manner in which data are retrieved. The level of detail required by the Cancer Registry needs to be evaluated.


Br. J. Cancer (1992), 66, 552-554 © Macmillan Press Ltd., 1992

### An audit of the quality of cancer registration data

R. Lapham[1] & N.R. Waugh[2]

[1]*Department of Pathology and* [2]*Public Health Medicine, University of Dundee, Ninewells Hospital and Medical School, Dundee, DD1 9SY, UK.*

**Summary** The accuracy of cancer registration data in the East of Scotland (Tayside) Cancer Registry was audited by comparing 200 consecutive registrations (about 10% of the annual total) with the 'gold standard' of the Histopathology records. ICD codes were independently generated by a pathologist by examining final pathology reports and then compared to those codes given by the local cancer registrar. Discrepancies were graded by the pathologist and the epidemiologist according to severity. Major errors of coding were few. Minor and moderate differences in coding occurred because of the nature and structure of the coding system and the manner in which data are retrieved. The level of detail required by the Cancer Registry needs to be evaluated.

Population-based cancer registry data are used to monitor incidence of, and survival from, the many types of cancer. Geographical differences in incidence stimulate thoughts about, and research into, the causes of cancer. However because some cancers are quite rare, the data must be highly accurate. A few cases misclassified could lead to apparently large differences in rates.

The Scottish Cancer Registration scheme had its origins in a system set up in 1936 by the National Radium Commission, and has developed over the years (S.H.H.D., 1990). Five regional registries feed information to the national one, based in the Information and Statistics Division of the Common Services Agency of the Scottish Health Service. The data can be used not only for monitoring incidence and survival, but by Health Boards assessing health care needs, evaluating the success of treatment, for economic appraisal of services, for assessing the representativeness of patients enrolled into clinical trials, for health education, where local information may have more impact than national rates, for record linkage to study the incidence of cancer in cohorts exposed to a possible carcinogen, and for assessing the effectiveness of cervical and breast screening programmes. Registration data have advantages over cancer mortality rates since survival rates can be calculated, and as the interval between exposure to a cause or causes, and incidence is less than that between exposure and mortality, retrospective enquiry should be easier.

Sources of information include Pathology and Haematology Departments, Radiotherapy Units, records staff in hospitals (through the cancer notification form, or from discharge summaries), General Register Office (by listing of deaths where cancer is mentioned), and various ad hoc sources, such as local specialist registers, screening programmes and research projects (S.H.H.D., 1990). The duplication of data from different sources helps ensure completeness of ascertainment, and the quality of data is thought to be very good.

However, one of the principles of audit suggested at the first meeting of the Tayside Medical Audit Committee, was that it was important to audit activities which were thought to be done well – nothing should be taken too much for granted.

The aim of this project was to examine the accuracy of registry data, by comparison with the 'gold standard' of Pathology reports, and with clinical case notes when required.

Correspondence: N.R. Waugh, Department of Public Health Medicine, Tayside Health Board, Vernonholme, Riverside Drive, Dundee, DD1 9NL, UK.

Received 1 August 1991; and in revised form 24 April 1992.

The East of Scotland registry records around 2,200 new cancers per annum. It is not at present computerised, and has three files, one by patient in alphabetical order, one by tumour, and one in registration order. Computerisation is eagerly awaited, and should happen late in 1991.

### Method

The names and Community Health Index numbers of 200 patients consecutively registered with the Tayside Regional Cancer Registry in early 1988, were extracted by the audit assistant. Pathology reports for these patients were then examined (if available) by the pathologist (RL) and the correct ICD 9 code for each cancer decided. Where there was no Pathology report (RL) examined the Hospital notes and decided the appropriate ICD 9 (WHO, 1977) classification. These clinician verified classifications were then compared with the codes allocated by the Cancer Registrar to identify cases where the codes differed. Discrepancies were classified in three grades:

*Minor* where there were differences in precise localisation.

*Moderate* where there was a misclassification of particular category of neoplasm within the main classification; sometimes such errors arose because of the archaic terminology of the ICD 9 code (e.g. continued use of terms 'lymphosarcoma' and 'reticulosarcoma').

*Serious* where a major misdiagnosis was given by Cancer Registry such as omission of second primary or allocation to an incorrect region (e.g. appendix adenocarcinoma attributed to rectum).

The reasons for the discrepancies were then examined. The Director of Cancer Registry then considered whether the differences would have significant effects on the epidemiological functions of cancer registration, and reclassified discrepancies accordingly.

### Results

The pathologist (RL) had access to histopathology surgical reports and post mortem reports that were carried out by the Department of Pathology Ninewells Hospital. This included surgical specimens received and post mortems of patients from Tayside Health Board including Ninewells Hospital, Dundee Royal Infirmary, Stracathro Hospital and Royal Victoria Hospital.

The surgical reports and post mortem reports were examined for details of site of tumour and type of tumour as required for ICD coding. Out of the 200 cases, 186 of the cases had corresponding pathology reports. In 11 of the cases hospital notes were required to confirm site and type of tumour. In three cases neither hospital notes nor pathology reports were available.

The ICD codes allocated by the reviewing pathologist (RL) were then compared with those given by the Cancer Registrar.

Of the cases examined, 145 were assigned the same ICD code by (RL) and the Cancer Registrar. Of these, 137 were confirmed by Pathology reports, eight were confirmed by Hospital notes.

The other 52 cases were assigned different ICD codes by (RL) and the Cancer Registrar of which (RL) sited 35 cases with minor differences, 6 with moderate differences and 11 with serious differences.

Of the minor differences, 29 cases were given different ICD codes due to minor differences in site of tumour (Tables I and II). Although the organ site was correct, the location within the organ differed (e.g. lung *vs* upper lobe, middle lobe, lower lobe). These differences sometimes occurred because more detailed information was available to (RL) from the Pathology report (e.g. Pathology reports gave gross descriptions of large surgical specimens or detailed localisation of tumours in necropsies which were not ascertained by the Cancer Registrar). However in other cases the Cancer Registry received more clinical information and so was more reliable or in other cases it relied on registration from concurrent cytology reports. In this Audit more information and therefore more accurate ICD codes were given by (RL) in 19 cases and by the Cancer Registrar in 9 cases. Six other minor discrepancies were also found, but these probably occurred because additional information was subsequently made available to the Cancer Registrar (Table III). Moderate differences in ICD coding were found in six cases. Four of these were differences in classification of Lymphoma. One Well Differentiated Lymphocytic Lymphoma was registered as Lymphosarcoma by the Cancer Registrar, and one Diffuse High Grade Immunoblastic Lymphoma was registered as a Reticulosarcoma. Two Nodular Sclerosing Hodgkin's Disease were registered as Unspecified Hodgkin's Disease. This type of discrepancy is a direct result of ICD lagging behind current medical practice terminology.

Two of the moderate differences relate to site. The Pathology report was not clear as to the type of tumour however the Cancer Registrar had given a definitive site ICD code. The category for carcinoma unspecified site was not used in these two cases (e.g. (RL) -bronchus? large cell neoplastic or renal metastasis, Cancer Registrar -bronchus carcinoma; (RL) -liver moderately differentiated adenocarcinoma? metastasis or 1° cholangiocarcinoma, Cancer Registrar -liver secondary carcinoma).

Eleven differences considered serious by the Pathologist were found. One of these was a case in which (RL) felt there were two different neoplasms requiring separate ICD codes but only one was given by the Cancer Registrar. (e.g. (RL) -Prostate Adenocarcinoma and Prostatic Urethra Transitional Cell Carcinoma; Cancer Registrar -Prostatic Adenocarcinoma only).

One case differed in coding due to Behaviour of tumour as (RL) felt Ovary Borderline Mucinous Cystadenocarcinoma should be registered under Neoplasm of Uncertain Behaviour.

Eight of the serious differences occurred because of spurious differences in site (Table IV).

In one case neither Pathology reports or Hospital notes showed any evidence of a neoplasm, but the Cancer Registrar gave an ICD code for Carcinoma of Unspecified type (Carcinomatosis).

Epidemiological review showed that of the 35 minor differences, only the nine cases with differences of site within colon (e.g. caecum versus colon unspecified) are of possible significance, since it may be that the aetiology of cancer of one part of the colon differs from that of another.

Of the moderate differences (six), the four lymphoma cases may matter, since such tumours are not common. In Tayside, the average numbers of cases per annum are:-

Hodgkin's – 10
Other lymphoid and histiocytic tissue – 28
Lymphosarcoma and reticulosarcoma – 20

It was felt therefore that a few misclassified cases could affect apparent incidence, and that this was an area of concern.

**Table I** Minor differences

| *Site* | *Number of cases with different codes assigned by (RL) and Cancer Registrar* |
|---|---|
| Lung or bronchus | 9 |
| Colon | 9 |
| Stomach | 4 |
| Female breast | 3 |
| Kidney | 1 |
| Larynx | 1 |
| Brain | 1 |
| Skin | 1 |

**Table II** Minor differences

| *RL* | *Cancer Registrar* |
|---|---|
| Main bronchus (2 cases) | Bronchus and lung, unspecified |
| Upper lobe (2 cases) | Bronchus and lung, unspecified |
| Bronchus and lung unspecified | Main bronchus (3 cases) |
| Bronchus and lung unspecified | Upper lobe |
| Bronchus and lung unspecified | Lower lobe |
| Transverse colon | Abdomen |
| Transverse colon | Colon, unspecified |
| Sigmoid colon | Colon, unspecified |
| Caecum (3 cases) | Colon, unspecified |
| Ascending colon | Colon, unspecified |
| Ascending colon | Caecum |
| Other, colon | Colon, unspecified |
| Cardia | Stomach, unspecified |
| Lesser curvature, unspecified | Stomach, unspecified |
| Other, stomach | Stomach, unspecified |
| Breast, unspecified | Upper inner quadrant breast |
| Breast, unspecified | Lower inner quadrant breast |
| Other, breast | Breast, unspecified |
| Renal pelvis | Kidney, except pelvis |
| Glottis | Larynx, unspecified |
| Brain, unspecified | Cerebellum |
| Skin of trunk | Skin of lower limb |

**Table III** Minor differences (not true errors)

| *RL* | *Cancer Registrar* |
|---|---|
| Omentum secondary neoplasm | Gastro-oesophageal primary |
| Pleura and liver secondary | Lung primary |
| Submandibular lymph node secondary | Pharynx primary |
| Omentum secondary | Stomach primary |
| Tonsil secondary | Floor of mouth primary |
| Skin secondary | Kidney primary |

**Table IV** Serious differences

| *RL* | *Cancer Registrar* |
|---|---|
| Appendix primary adenocarcinoma | Rectum adenocarcinoma |
| Omentum secondary carcinoma | Omentum primary carcinoma |
| Skin (L) ear, (R) cheek squamous carcinoma (2 ICD codes) | Skin (R) ear, (R) carcinoma cheek (1 ICD code) |
| Liver secondary carcinoma | Liver primary carcinoma |
| Maxilla primary carcinoma | Antrum (stomach) primary carcinoma |
| Prostate – carcinoma arising urethra epithelium | Prostatic carcinoma |
| Pancreas ? Metastasis | Pancreas primary |
| Jaundice ? Pancreatic carcinoma (from notes) | Pancreas primary |

The serious differences (11) were composed mainly of two categories, firstly secondaries classed as primaries, and secondly wrong sites. The latter were too few to affect incidence rates, and hence are not of epidemiological significance. The former could be significant in cases such as liver, where primary tumours are uncommon (usually around 15 per annum in Tayside). Hence two cases respectively misclassified could increase reported incidence by 13%.

### Discussion

Fifty two out of 200 cases with different codes represents a fair proportion of differences between (RL) and the Cancer Registrar. At most eight of these (4% of total) represent serious differences in which a site was coded wrongly. Problems inherent in the structure of the audit account for a few of the minor differences in ICD codes as (RL) was restricted to pathology reports and examined hospital notes only in cases when pathology notes were not available.

Numerically the large bulk of minor differences (29) and moderate differences have occurred because of the nature of the ICD coding and the manner in which data is retrieved. The pathology reports are an easy and reliable way to obtain data for registration purposes and as this study shows 186 out of 200 cases examined had pathology reports. They have reliable data as to tumour type. The site however, in certain cases, tends to be less well defined. In biopsy specimens this data is supplied on the request form by the clinician and is usually not as detailed as the ICD coding requires (e.g. exact site on lip – upper lip vermilion border, lower lip vermilion border, upper lip inner aspect, lower lip inner aspect, lip unspecified inner aspect, commissure of lip, other, lip unspecified vermilion border). The exact site is often not necessary to the pathologist in making his disease category diagnosis. If an entire specimen is received the detailed information as to site within an organ may be in the pathology report gross description but this may not be easily inferred by secretarial staff transferring information to be registered. Perhaps a more simplified coding to site should be considered as the current method of coding results in data with many inaccuracies in detailed site within an organ, and certainly an abundance of ICD codes given with an unspecified site within an organ. Otherwise more time consuming and laborious consultation of hospital notes and canvassing of clinical data would be required for more accurate coding. If computerisation of cancer registration places more reliance on pathology reports spurious inaccuracies will be created by lack of exact sites being quoted on request forms. The Cancer Registrar's access to a range of other data sources can improve data quality.

Another main category that contains inadequacies in its format are those codes concerning primary neoplasms of lymphatic and haematopoietic tissue. This includes sections 'Lymphosarcoma and Reticulosarcoma, Hodgkin's disease, and other malignant neoplasms of lymphoid and histiocytic tissue'. The classification of lymphomas and lymphoproliferative disorders is a vast and specialised field. The ICD coding system makes attempts at categorising some of these neoplasms and disorders. The Cancer Registrars receive some training in assigning ICD codes but in such a rapidly changing and complicated subject it is optimistic to suggest a structured coding for lymphomas in this setting. This audit revealed two cases of lymphoma that were categorised under Reticulosarcoma and Lymphosarcoma. This terminology is now outdated and their meaning is obscure in light of more advanced terminology. The Hodgkin's diseases are coded by two subclassifications, the Parker Jackson classification and the Rye modification of Lukes–Butler. The Parker Jackson classification dates from 1947 and again is outdated and redundant terminology that should be discarded. The Rye modification of Lukes–Butler is presently a good standard classification but as this audit showed two cases of Nodular Sclerosing Hodgkin's disease were classified as Unspecified Hodgkin's disease (Robb-Smith & Taylor 1981).

Other similar difficulties are present in the categories of 'other malignant neoplasms of lymphoid and histiocytic tissue'. These problems highlight again that errors are produced by complicated categories.

This audit shows that major errors of coding are few, however there are numerous differences in coding between the auditor and the Cancer Registrar that occurred because of the structure and age of the ICD classification, and the way information is obtained. The Cancer Registry needs to consider the level of detail required. If exact sites are necessary then more meticulous collection of clinical data will be required to avoid unspecified or wrong sites being quoted. If automatic download of information from the histopathology computer system becomes the main source of cancer registry data, it will need to be supplemented from other sources unless clinicians can be persuaded to improve on request forms.

The problems of outdated ICD classification are more difficult to overcome. This audit was carried out towards the end of the life of ICD 9. It is probably unrealistic to expect any classification which can be revised only every 10–15 years to keep pace with advances in medical science, and since the implementation of each revision creates considerable problems for records staff, statisticians and epidemiologists, more frequent revision would not be welcome.

For many purposes, summarised cancer registration data are adequate, and the discrepancies noted here are not important. It is probably only when a rare tumour is being studied, that they are of epidemiological significance. For example, at present there is considerable interest in incidence of lymphomas and leukaemias in areas close to nuclear installations. However in such studies (Heasman *et al.*, 1987; Roman *et al.*, 1987) it is usual for diagnosis to be verified from both clinical and pathological records. It would be wise for this to continue.

In conclusion, the quality of cancer registration data is good, but could be improved. Since we completed this study, the Scottish Cancer Registration Organisation has held a study day for cancer registrars and others, on the topic of classification and registration of lymphomas and leukaemias. Further improvements should follow computerisation of the registry, which will permit much greater use of the data, including feedback to clinicians, who will be encouraged to comment on possible inaccuracies. Computerisation elsewhere – for example in Radiotherapy, Haematology and Gynaecology – will also help by providing lists of patients with specific cancers, which can be used to check completeness of registry ascertainment.

We thank Mrs M. Cannon, Cancer Registrar, and Mrs G. Alexander, Medical Audit Assistant for extracting registrations, and Professor J. Swanson Beck for commenting on the first draft.

### References

HEASMAN, M.A., URQUHART, J.E., BLACK, R.J., KEMP, I.N., GLASS, S. & GRAY, M. (1987). Leukaemia in young persons in Scotland: a study of its geographical distribution and relationship to nuclear installations. *Health Bull.*, **45**, 147–151.

ROBB-SMITH, A.H.T. & TAYLOR, C.R. (1981). *Lymph Node Biopsy*. Miller Heyden: London.

ROMAN, E., BERAL, V., CARPENTER, L., WATSON, A., BARTON, C., RYDER, H. & LYNN ASTON, D. (1987). Childhood leukaemia in the West Berkshire and Basingstoke and North Hampshire District Health Authorities in relation to nuclear establishments in the vicinity. *Br. Med. J.*, **294**, 597–602.

SCOTTISH HOME AND HEALTH DEPARTMENT. (1990). *Review of the Scottish Cancer Registration System*. Edinburgh.

WORLD HEALTH ORGANISATION. (1977). *International Classification of Diseases* (9th revision). Geneva.